# Reduced bacterial mortality and enhanced viral productivity during sinking in the ocean

**DOI:** 10.1038/s41396-022-01224-9

**Published:** 2022-04-01

**Authors:** Wei Wei, Xiaowei Chen, Markus G. Weinbauer, Nianzhi Jiao, Rui Zhang

**Affiliations:** 1 College of the Environment and Ecology, Xiamen University, Xiamen, 361102 PR China; 2 State Key Laboratory of Marine Environmental Science, Fujian Key Laboratory of Marine Carbon Sequestration, Xiamen University, Xiamen, 361102 PR China; 3 School of Environmental Ecology and Biological Engineering, Wuhan Institute of Technology, Wuhan, 430205 PR China; 4 College of Ocean and Earth Sciences, Xiamen University, Xiamen, 361102 PR China; 5 Sorbonne Universités, UPMC, Université Paris 06, CNRS, Laboratoire d’Océanographie de Villefranche (LOV), Villefranche-sur-Mer, 06230 France; 6 Southern Marine Science and Engineering Guangdong Laboratory (Zhuhai), Zhuhai, 519080 PR China

**Keywords:** Microbial ecology, Microbial ecology

## Abstract

Particle sinking is an important process in the ocean, influencing the biogeochemical cycle and driving the long-term preservation of carbon into the deep sea via the biological pump. However, as an important component of marine ecosystems, the role of viruses during sinking is still poorly understood. Therefore, we performed a series of transplantation experiments in the South China Sea to simulate environmental changes during sinking and investigate their effects on viral eco-dynamics and life strategy. Our study demonstrated increased viral production but decreased virus-mediated bacterial mortality after transplantation. A larger burst size and switch from the lysogenic to lytic strategy were shown to contribute to enhanced viral productivity. We provide experimental evidence that surface viral ecological characteristics changed dramatically after transplantation into deep-sea waters, indicating a potential importance of viruses during vertical sinking in the ocean. This effect probably provides positive feedback on the efficiency of the biological pump.

## Introduction

The vertical sinking of particles is one of the most important mechanisms driving oceanic dynamics throughout physical, chemical, and biological processes on a global scale. It is a major channel to transport and redistribute fixed carbon through photosynthesis from the sea surface to the deep ocean [1]. As a key component of the “biological pump” (BP) in the ocean, the particulate organic matter (POM) produced in the photic zone aggregates by a complex interplay among faecal pellets, carcasses of zooplankton, phytoplankton cells, and prokaryotic cells [2, 3] and regulates the global climate by long-term sequestration of atmospheric CO_2_ [4]. For instance, *ca*. 1–40% of the photosynthetically fixed CO_2_ flows into the dark zone of the ocean in the form of sinking POM [5]. Moreover, the sinking POM is continuously degraded and releases the dissolved organic matter (DOM) and nutrients, which are used by microbial communities and made available for recycling into the microbial loop [6]. As the main microbial communities on POM, colonizing bacteria have been revealed to harbor distinct features regarding diversity, structure, and dynamics, which are significantly different from those of the surrounding free-living bacterioplankton [7–9]. They are considered an important transformer in driving the disaggregation and remineralization of sinking POM to regulate the efficiency of the BP [10–13], and play a substantial role in global biogeochemical cycling [14, 15].

Over the past three decades, viruses have been found to be the most abundant “life forms” in global oceans, playing an important ecological role [16, 17]. They infected bacteria and are responsible for high bacterial mortality [18, 19]. Thus, the “viral shunt” could enhance the transformation of POM (e.g., host cells) into DOM (e.g., nucleic acid and protein) [20] and subsequently impact biogeochemical cycling in the global oceans [6, 17, 21, 22]. During sinking, viruses can be transplanted into the deep ocean directly through adsorption on POM or indirectly through infected cell hosts (e.g., viral particles in the infected bacterial cells) [23, 24]. However, up to present, the bacterium-virus interaction and the role of viruses in these processes are unclear. A recent study found that the transferred viral particles still retain infectivity for years in the deep-ocean environment [25], indicating that the vertical transportation of these viruses may continuously contribute to the activity of the deep-ocean viral population [26, 27]. Thus, we hypothesize that such direct and indirect vertical transportation in the ocean could impact the virus-host interaction, and subsequently influence the ecological role of microbes in the sinking process, as well as the efficiency of the BP.

**Fig. 1:**
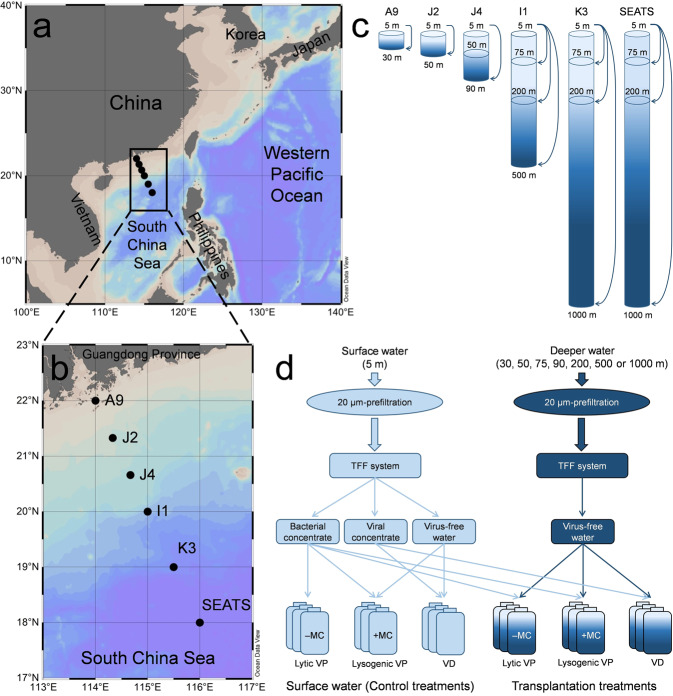
Map of sampling stations and the design of transplantation experiments in the South China Sea. The seawater samples were collected from the total 19 layers of the six stations (**a**, **b**, generated by Ocean Data View 4 software, https://odv.awi.de/). The transplantation experiments of surface bacterial/viral concentrate (5 m depth) mixed with virus-free seawater of each deeper layer were performed at the six stations (**c**, **d**). The schematic diagram of the transplantation experiments is shown (**d**) and detailed in the text. Abbreviations represent tangential flow filtration (TFF), incubation with mitomycin C (+MC), incubation without mitomycin C (–MC), viral production (VP) and viral decay (VD).

To verify our hypothesis and better understand how the vertical transportation affects virus-host interactions by altering viral activity, dynamics and life strategy, we performed 18 transplantation experiments at 6 stations in the South China Sea (SCS) (Fig. 1). Our study for the first time reveals the effect of vertical transplantation on viral eco-dynamics and life strategies, such as the lytic and lysogenic viral production rate (VP_R_), viral decay rate (VD_R_), burst size (BS), virus-mediated mortality (VMM) and fraction of lysogenic cells (FLC), and highlights the importance of viruses in vertical sinking in the ocean, which may influence the efficiency of the BP.

## Materials and methods

### Study area, sampling, and environmental parameters

Seawater samples were obtained at six stations along the Pearl River Estuary to the pelagic area of the SCS during a research cruise from August to September 2014 (Fig. 1a, b). The sampling covered three typical pelagic zones: upper epipelagic (0–75 m), lower epipelagic (75–200 m), and mesopelagic ocean (200–1000 m). At each station, water samples were collected with a CTD (SBE9/11 plus, Sea-Bird Electronics, Inc., USA) rosette sampler equipped with 12 Niskin bottles (12 l each) and then prefiltered with 20-µm mesh filters to remove zooplankton. Although this operation removed particles larger than 20 µm, previous investigations showed that these large POM aggregates occupy relatively small fraction (*ca*. 0.1%) in total particles in surface ocean [28, 29]. The prefiltered water was prepared for the determinations of picoplankton and virioplankton abundances and the following viral dynamics and life strategy experiment. The CTD was also equipped with sensors for measuring in situ data of depth, temperature, and salinityin each water layer of the stations. In addition, samples (500 ml) for nutrient analysis were stored at –20 °C, and then the concentrations of ${{{{{{{\mathrm{NO}}}}}}}}_3^--$ + ${{{{{{{\mathrm{NO}}}}}}}}_2^--$, ${{{{{{{\mathrm{NO}}}}}}}}_2^--$, ${{{{{{{\mathrm{SiO}}}}}}}}_3^{2-}$ and ${{{{{{{\mathrm{PO}}}}}}}}_4^{3-}$ were determined by an Auto Analysis III, AA3 instrument (Bran-Luebbe, Germany) in the laboratory.

### Picoplankton and virioplankton abundance

The subsamples (1.98 ml of seawater) for picoplankton and virioplankton abundance measurement were fixed with glutaraldehyde (20 µl, Sangon) at a final concentration of 0.5%. After incubation at 4 °C for 15 min in the dark, they were flash-frozen in liquid nitrogen and then stored at −80 °C until analysis [30]. The abundances of picoeukaryotes, *Synechococcus*, *Prochlorococcus*, heterotrophic bacteria, and viruses were measured by using a flow cytometer (FCM, Epics Altra II, Beckman Coulter, USA) equipped with an air-cooled laser with a wavelength of 488 nm (Sapphire, Coherent, USA). Specifically, the samples for virus counting were diluted in Tris–EDTA buffer (pH: 8; Sigma), stained with SYBR Green I (0.5 × 10^−4^ dilution of the commercial stock solution; Molecular Probes), incubated for 10 min at 80 °C in a thermostat water bath (DKB-501A, Shanghai Jinghong, China), and then cooled to room temperature for FCM analysis [31, 32]. Heterotrophic bacterial counting was performed on samples that were incubated with SYBR Green I (1 × 10^−4^ dilution of the commercial stock solution) for 15 min in the dark. In addition, the abundances of picoeukaryotes, *Synechococcus*, and *Prochlorococcus* were directly determined by FCM without staining [33]. For all these samples, a suspension of yellow-green fluorescent beads (1 µm in diameter; Molecular Probes) was added as an internal standard, and all data were analysed with FCS Express V3 software (De Novo Software, http://www.denovosoftware.com/). The typical scatter plots of each microbial group were shown in Fig. S1 and additional details about the FCM analysis and data treatment can be found in the previous reports [34, 35].

### Lytic and lysogenic viral production

Lytic and lysogenic VP was measured through the dilution approach [36] for samples from the different depth layers and transplant experiments. Fifty milliliters of the bacterial concentrate obtained through tangential flow filtration (TFF) with a 0.22-μm polyvinylidene difluoride (PVDF) cartridge (Labscale, Millipore) from 600 ml of in situ seawater was mixed with 250 ml of virus-free filtrate produced from the same water sample obtained through TFF with a 30 kDa molecular weight polysulfone cartridge (Labscale, Millipore). This operation made viral abundance decline to *ca*. 10–20% of the initial abundance, and the bacterial concentration was similar to the in situ status [37]. Finally, the experiments measuring lytic and lysogenic VP were performed in 50 ml aseptic tubes (duplicate) with and without adding 1 µg ml^–1^ mitomycin C (final concentration; Roche) [20, 38], which were then incubated in dry bath incubators (MK-20, Hangzhou Allsheng, China) at in situ temperature in the dark. Subsamples (1 ml) were collected to enumerate the viral abundance at 0, 3, 6, 9, and 12 h of incubation. The lytic and lysogenic VP (viruses ml^–1^ h^–1^) was estimated through viral accumulation in each 12 h incubation with the VIPCAL online program (http://www.univie.ac.at/nuhag-php/vipcal/) [39]. Furthermore, the VP_R_ was calculated as ${{{{{{{\mathrm{VP}}}}}}}}_R\left( {{{{{{{{\mathrm{\% }}}}}}}}h^{{{{{{{{\mathrm{-1}}}}}}}}}} \right) = \frac{{{{{{{{{\mathrm{VP}}}}}}}}\left( {{{{{{{{\mathrm{viruses}}}}}}}}\;{{{{{{{\mathrm{ml}}}}}}}}^{{{{{{{{\mathrm{-1}}}}}}}}}{{{{{{{\mathrm{h}}}}}}}}^{{{{{{{{\mathrm{-1}}}}}}}}}} \right)}}{{{{{{{{{\mathrm{VA}}}}}}}}_0}} \times {{{{{{{\mathrm{100\% }}}}}}}}$, where VA_0_ represents the viral abundance at 0 h in the VP experiment. The BS was defined as ${{{{{{{\mathrm{BS}}}}}}}} = \frac{{{{{{{{{\mathrm{VP}}}}}}}}({{{{{{{\mathrm{viruses}}}}}}}}\,{{{{{{{\mathrm{ml}}}}}}}}^{-1}h^{-1})}}{{\Delta {{{{{{{\mathrm{BA}}}}}}}}({{{{{{{\mathrm{cells}}}}}}}}\;{{{{{{{\mathrm{ml}}}}}}}}^{-1}h^{-1})}}$, where ΔBA represents the amount of bacterial death determined from the reduction in bacterial abundance per hour during the incubation of the VP experiments, and is calculated by using the equation $\Delta {{{{{{{\mathrm{BA}}}}}}}} = \left( {\frac{{{{{{{{{\mathrm{BA}}}}}}}}_{{{{{{{{\mathrm{max}}}}}}}}\;1}-{{{{{{{\mathrm{BA}}}}}}}}_{{{{{{{{\mathrm{min}}}}}}}}\;1}}}{{t_{{{{{{{{\mathrm{min}}}}}}}}\;1}-t_{{{{{{{{\mathrm{max}}}}}}}}\;1}}} + \ldots + \frac{{{{{{{{{\mathrm{BA}}}}}}}}_{{{{{{{{\mathrm{max}}}}}}}}\;{{{{{{{\mathrm{n}}}}}}}}}-{{{{{{{\mathrm{BA}}}}}}}}_{{{{{{{{\mathrm{min}}}}}}}}\;{{{{{{{\mathrm{n}}}}}}}}}}}{{t_{{{{{{{{\mathrm{min}}}}}}}}\;{{{{{{{\mathrm{n}}}}}}}}}-t_{{{{{{{{\mathrm{max}}}}}}}}\;{{{{{{{\mathrm{n}}}}}}}}}}}} \right)/n$. This formula for BS calculation is an improved method according to prior studies [40, 41], where BA_max n_ and BA_min n_ represent the maximum and minimum bacterial abundance at the n^th^ peak of the polyline with bacterial abundance versus time (i.e., 0, 3, 6, 9, and 12 h) in the VP experiment, and t_max n_ and t_min n_ are the corresponding times. Since grazers were destroyed or inactivated by tangential flow filtration [37], the bacterial mortality in the incubation system is mainly due to viral lysis in our study. Therefore, the VMM was calculated by using the equation ${{{{{{{\mathrm{VMM}}}}}}}} = \frac{{\Delta {{{{{{{\mathrm{BA}}}}}}}}}}{{{{{{{{{\mathrm{BA}}}}}}}}_0}} \times 12{{{{{{{\mathrm{h}}}}}}}}$, where BA_0_ represents the 0 h bacterial abundance in the incubation of the VP experiment. In addition, FLC, i.e., the percent of lysogenic cells, was estimated by using the formula reported in the VIPCAL online program (http://www.univie.ac.at/nuhag-php/vipcal/) [39].

### Viral decay

The VD_R_ was measured through the reported approach [42]. Sample water was filtered through a 0.22-μm PVDF cartridge (Labscale, Millipore) in 50 ml aseptic tubes (duplicate) and incubated in dry bath incubators (MK-20, Hangzhou Allsheng, China) at in situ temperature in the dark. Subsamples (1 ml) were collected at 0, 3, 6, 9, and 12 h of incubation, and the viral abundances were determined at each time point by using FCM. We calculated the VD_R_ by fitting a linear regression to the decline in ln-transformed viral abundance versus time in the incubation (see Fig. S4 in Wei et al. [41] as an example). Therefore, the slope of the line multiplied by 100 was the decay rate and was expressed as a percentage per hour [42].

### Experimental setup of transplantation

The bacterial/viral concentrate obtained from the 5 m depth was mixed with virus-free seawater of each deeper layer at the six stations (Fig. 1c), for example, in Station J4 with four experiments (Fig. 1d): (1) 5 m bacterial concentrate + 5 m virus-free seawater with/without mitomycin C (in situ lysogenic/lytic VP); (2) 5 m viral concentrate + 5 m virus-free seawater (in situ VD); (3) 5 m bacterial concentrate + 90 m virus-free seawater with/without mitomycin C (transplanted lysogenic/lytic VP); and (4) 5 m viral concentrate + 90 m virus-free seawater (transplanted VD). Each sample was added into a 50 ml aseptic tube (duplicate) and incubated in a dry bath incubator for 12 h with the in situ temperature of each layer and in the dark to measure the (lytic and lysogenic) VP and VD as described above. Similar to the dilution approach for measuring VP_R_ [36], the mixture of viral concentrate (50 ml) with each deeper layer of virus-free water (250 ml) was used to determine the VD_R_ in the transplant experiments.

### Statistical analysis

Using the SPSS (19.0) software package (SPSS Inc., Chicago, IL, USA), Pearson’s correlation analysis was applied to assess the degree of correlation among the biotic or abiotic parameters investigated, and Student’s *t*-test (paired design) was performed to assess the significant difference in the (lytic and lysogenic) VP_R_, VD_R_, BS, FLC and VMM between surface water and transplantation treatments. A *p* value < 0.05 was used to indicate statistical significance. In addition, distance-based multivariate analysis for a linear model using forward selection (DISTLM-*forward*) was used to test the relationships between viral and environmental parameters in Primer 6 software with the PERMANOVA + package (Primer-E, Plymouth, United Kingdom).

## Results and discussion

### In situ environmental and microbial parameters

The in situ environmental variables at the six stations of the SCS with a total of 19 layers were investigated, covering the typical regions of the SCS, from coastal to offshore sea. The temperature ranged from 4.4 to 29.8 °C and dropped sharply with increasing depth (Fig. S2 and Table S1). The salinity content tended to increase rapidly from the surface of the coastal sea to the deep layer of the offshore sea, varying from 29.6 to 34.6 (Fig. S2 and Table S1). Similarly, the concentrations of ${{{{{{{\mathrm{NO}}}}}}}}_3^--$+${{{{{{{\mathrm{NO}}}}}}}}_2^--$, ${{{{{{{\mathrm{SiO}}}}}}}}_3^{2-}$ and ${{{{{{{\mathrm{PO}}}}}}}}_4^{3-}$ in the SCS displayed a trend showing that the deep water contained higher nutrient versus surface water, with mean values of 12.10 ± 16.32 μmol l^–1^, 22.38 ± 34.23 μmol l^–1^ and 1.26 ± 2.15 μmol l^–1^, respectively (Fig. S3 and Table S1).

The autotrophic microbes decrease in abundance with depth at three stations at open sea. Specifically, the *Synechococcus*, *Prochlorococcus* and eukaryotic abundances varied from 3.31 ± 0.25 × 10^5^ cells ml^–1^ to 1.04 ± 0.09 × 10^4^ cells ml^–1^, 1.08 ± 0.03 × 10^5^ cells ml^–1^ to 9.10 ± 4.30 × 10^1^ cells ml^–1^ and 6.54 ± 0.69 × 10^3^ cells ml^–1^ to 1.50 ± 3.60 × 10^1^ cells ml^–1^, respectively (Table S1). The heterotrophic bacterial and viral abundances were 7.23 ± 5.44 × 10^5^ cells ml^–1^ and 6.41 ± 4.24 × 10^6^ viruses ml^–1^ on average, respectively, and tended to increase with depth at Stations A9 and J2 and decrease with depth at the other four stations. Horizontally, the heterotrophic bacterial abundance decreased from the coastal (1.77 ± 0.07 × 10^6^ cells ml^–1^) to the offshore area (7.21 ± 0.02 × 10^5^ cells ml^–1^) at the surface, while the viral abundance showed a maximum of 1.01 ± 0.01 × 10^7^ viruses ml^–1^ at Station J4.

Vertically, the lytic VP_R_ tended to increase with depth at five of the six stations, and the lysogenic VP_R_ increased with depth at Stations J2, K3, and SEATS but tended to decrease with depth at Stations A9, J4, and I1. Horizontally, the lytic VP_R_ tended to increase from the coast to the offshore area at the surface, while the lysogenic VP_R_ did not show a clear trend. The VD_R_ showed a downward trend with depth at each station and a weak rising trend from the coastal to the offshore area at the surface, with a range from 0.58 ± 0.10% h^–1^ (30 m at Station A9) to 4.14 ± 0.50% h^–1^ (5 m at Station I1). Overall, the distribution of microbiological parameters was consistent with previous studies in the SCS and other marine systems [31, 32, 42–48].

The virus-to-bacterium ratio (VBR) is an index to represent the relationship between viruses and bacteria and usually reflects the balance of VP and VD [49, 50]. In our investigation, the VBR ranged from 4.78 to 23.97 and was higher in the deeper layers of water than in the surface water (9.45 ± 1.35 vs. 9.15 ± 1.10 on average). However, there was no significant correlation between VBR and lytic VP_R_, lysogenic VP_R_ and VD_R_ in our investigation (Pearson’s correlation analysis, data not shown). We further found that only 57% of the total variation in the VBR was explained by viral abundance, ${{{{{{{\mathrm{PO}}}}}}}}_4^{3-}$ concentration and *Synechococcus* abundance (DISTLM analysis, Table S2). These suggest that additional environmental processes may strongly regulate the distribution of the VBR. In addition to viral particles originating from the in situ deep ocean, they can be continuously transported from surface water through several potential mechanisms. First, viral particles are transported into the deep ocean through adsorption on POM [23]. Second, infected cells were inevitably adsorbed by POM to transfer into the deep ocean, indirectly resulting in the sinking of numerous viral particles [24, 51]. Additionally, internal waves, mesoscale eddies, and other processes promote the mixing of water masses [52–54], driving the potentially vertical mixing of bacterioplankton and virioplankton. Therefore, the relatively high VBR in the deep ocean may be explained by viruses sinking from surface water [50, 55, 56]. Other factors influencing VBR are discussed further down.

### Vertical transplantation affecting viral production and viral decay rates

**Fig. 2:**
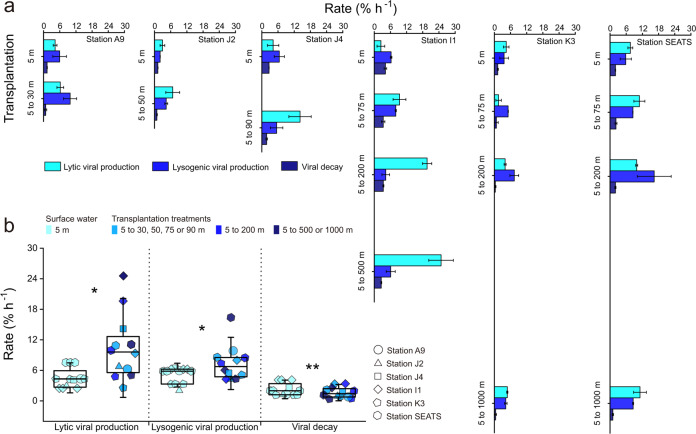
Viral activity in the transplantation experiments. **a** Lytic and lysogenic viral production and viral decay in control and transplantation experiments performed in six stations. Error bars indicate the standard errors calculated from duplicate sample measurements. **b** Comparison of lytic and lysogenic viral production and viral decay in transplantation experiments with control treatments. Error bars indicate the standard deviations calculated from all measurements in control or in transplantation experiments. ns, no significant difference; *p < 0.05; **p < 0.01.

The lytic VP_R_ of the transplantation treatments (i.e., the surface bacterial concentrate transplanted into virus-free seawater of each deeper layer) at each station was significantly higher than that of the surface water (*n* = 12, *p* < 0.05), at 9.21 ± 4.92% h^–1^ vs. 3.81 ± 1.66% h^–1^ on average (Fig. 2a, b). This increasing trend was most obvious at Station I1, where the lytic VP_R_ increased with *ca*. an order of magnitude from the surface water (2.47 ± 2.02% h^–1^) to the deepest layer transplantation treatments (24.78 ± 6.43% h^–1^), and the lowest increase was observed at Station SEATS, which exceeded 17%. This result is unexpected since the viral abundance and production showed a relatively low level in the deep ocean in our study (Table S1) and other deep ocean environments, which could be explained by the low temperature, high pressure and limited input of exogenous organic carbon [46, 47, 56].

**Fig. 3:**
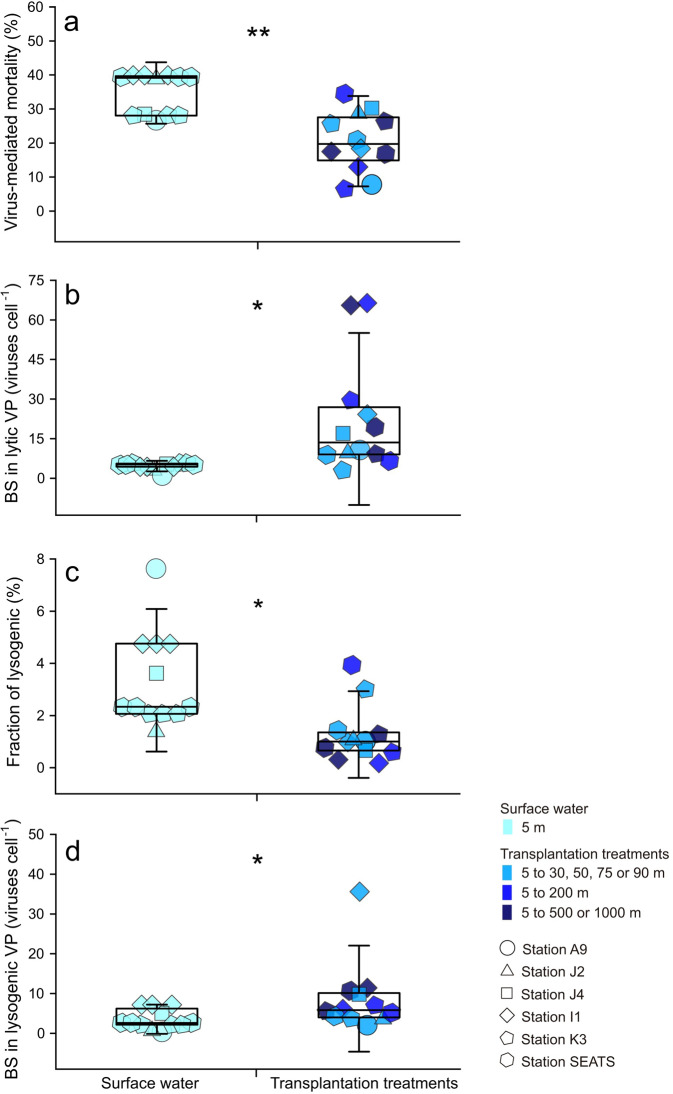
Viral impacts on bacterioplankton in the transplantation experiments. **a** Virus-mediated bacterial mortality. **b** Burst size of lytic viral production. **c** Fraction of lysogenic bacterial cells. **d** Burst size of lysogenic viral production. Error bars indicate the standard deviations calculated from all measurements in control or in transplantation experiments. BS burst size, VP viral production; ns, no significant difference; *p < 0.05; **p < 0.01.

Three possible reasons could result in an increase in lytic VP_R_ in the transplantation treatments: (1) increased host infection rate, (2) increased BS, and (3) switch from the lysogenic to lytic strategy. In the VP experiments, the production rate was estimated by the reduction and reoccurrence assay [36]. This dilution approach resulted in *ca*. an order of magnitude reduction in viral abundance, thus greatly decreasing the virus-host contact rate (i.e., blocking new infections) [37]. Therefore, the production of progeny viruses was likely not due to an enhanced host infection rate; rather it was due to the lysis of already infected cells. In fact, the VMM was lower in the transplantation treatments than in the surface water (*n* = 12, *p* < 0.01; Fig. 3a). This observation is most likely due to a decrease in temperature, which slows down intracellular viral replication in bacterial cells thus causing a delay of the latent period and a decline in VMM [57]. Interestingly, the BS was 3.89-fold higher in the transplantation treatments than in the surface water (Fig. 3b), which was consistent with the increase of BS in natural environment with depth [20]. An increase in BS implied that the host had increased metabolic activity during transplantation, which provided additional substances and energy for virus replication [51]. The composition of the viral particles is relatively rich in the elements of N and P [22], so viral replication in infected cells often is limited by the scarcity of P and N in oligotrophic surface oceans [58]. Inhibition of viral production was found during the incubation of both isolates and natural communities under the condition of nutrient limitation [59, 60]. Another study showed that the addition of inorganic nutrients increased the production rate of viral community by 14–52% in dark incubation [61]. Motegi and colleagues also suggested that addition of inorganic P and N can enhance prokaryotic growth and viral production in the surface ocean [58, 60]. Indeed, in 5 of 6 stations, the concentrations of ${{{{{{{\mathrm{NO}}}}}}}}_3^--$+${{{{{{{\mathrm{NO}}}}}}}}_2^--$, ${{{{{{{\mathrm{SiO}}}}}}}}_3^{2-}$ and ${{{{{{{\mathrm{PO}}}}}}}}_4^{3-}$ clearly increased with depth (Fig. S3). Therefore, when the surface bacteria were transplanted into the deep ocean, the bacterial activity could have been increased by surrounding sufficient nutrients, which in turn could facilitate the synthesis of progeny viruses in infected cells and increased VP [62]. In addition, our data showed that the increased BS offset the decline in VP due to reduced VMM and caused a general increase in lytic VP_R_.

The shift from lysogeny to lysis could have been an alternative or additional reason for increased lytic VP_R_ in the deeper-layer transplanted incubation. Lysogeny is a viral life strategy in which the viral genome integrates into the host’s genome to form prophages and initiate a symbiotic relationship with its host until the lytic cycle is induced [63]. Previous studies found that a high rate of lysogenic bacteria survived in conditions of low prokaryote abundance, activity, and production [49, 64]. In general, the lysogenic strategy has been reported to be dependent on changes in temperature, salinity and nutrient concentrations [49, 65–69], and environmental variation or shock was considered an inducing agent. For example, salt stress was found to shock the stability of bacteriophage λ^imm434^ lysogens [70]. ${{{{{{{\mathrm{PO}}}}}}}}_4^{3-}$ was considered as an important factor in the lysis-lysogeny switch by previous studies. For example, Tuomi et al. suggested that the increase in inorganic P concentration may induce the lysis of some lysogenic bacterial populations in dark incubation, resulting in the release of viral particles [71]. Therefore, when surface host cells were transplanted into deeper seawater, they experienced changes in environmental factors, e.g., lower temperature, higher salinity, and higher concentrations of inorganic nutrients. The lytic VP_R_ can be increased by the switch from viral lysogenic to lytic strategies, which release progeny virus particles. Indeed, the FLC was significantly lower in the transplantation treatments than in surface water, 1.31 ± 0.32% vs. 3.30 ± 0.53% (*n* = 12, *p* < 0.05; Fig. 3c), suggesting that environmental changes due to transplantation can trigger the switch from lysogeny to lysis and cause lysis afterward to release more viral particles. Furthermore, the lysogenic VP_R_ was significantly higher in transplantation treatments than in surface water (*n* = 12, *p* < 0.05), with a range of increase from 28.0 to 180.7% (Fig. 2a, b), which was also probably due to the increased BS when the lysogenic strategy switched to the lytic strategy in the transplantation treatments (*n* = 12, *p* < 0.05; Fig. 3d). Altogether, the results suggested that vertical sinking may enhance both lytic and lysogenic VP_R_ via the promotion of BS, probably due to additional nutrient supply, and trigger the switch from the lysogenic to lytic strategy, probably due to environmental changes.

In contrast to lytic and lysogenic VP_R_, the VD_R_ in the surface water was significantly higher than that in the transplantation treatments (*n* = 12, *p* < 0.01) and tended to decrease with the depth of transplantation (Fig. 2a, b). Comparing the data between surface water and deepest layer transplantation treatments, the strongest reduction was 59.1% at Station K3 and that the weakest reduction was 28.6% at Station J2. These results suggested that the VD_R_ was inhibited by sinking into the deep ocean, which may be mainly caused by the low temperature and low activity of extracellular enzymes [32, 42, 72]. Low temperature contributes to the stability of membrane lipids and the protein capsids of viral particles, e.g., it protects the biomolecular elasticity and molecular structure of proteins, thus reducing the degradation of viral particles [73]. In addition, when the surface virioplankton were transplanted into the deeper seawater, fewer extracellular enzymes due to less biomass produced and low temperature in the transplanted incubation could have decreased their activity [47, 74, 75], resulting in a reduction in VD_R_.

### Biogeochemical significance

Both bacteria colonized on POM and surrounding free-living bacteria are considered important drivers causing rapid degradation of the sinking POM [15, 76, 77]. Our study shows that viral particles continued to be produced by the lysis of infected bacteria in vertical transplantations, probably resulting in new infection and lysis events. Together with the low VD_R_ in the deep ocean, these viruses indirectly transmitted from surface water are an underlying mechanism contributing to the high VBR, a common phenomenon observed in deep-ocean environments, such as the North Atlantic Ocean [55], South Atlantic Ocean [78], and Pacific Ocean [79]. In addition, viral lysis occuring during particle sinking will regulate bacterial diversity and community struture and, subsequently, impact the biological utilization and transformation of both particular and dissolved organic matters. This indirect influence of viruses on quantity and quality of organic matters can be revealed by monitoring bacterial and viral community with molecular ecological techniques, distinguishing group-specific responses with cell sorting techniques [80, 81], and detecting taxon-specific lysis with polony and ipolony techniques [82].

**Fig. 4:**
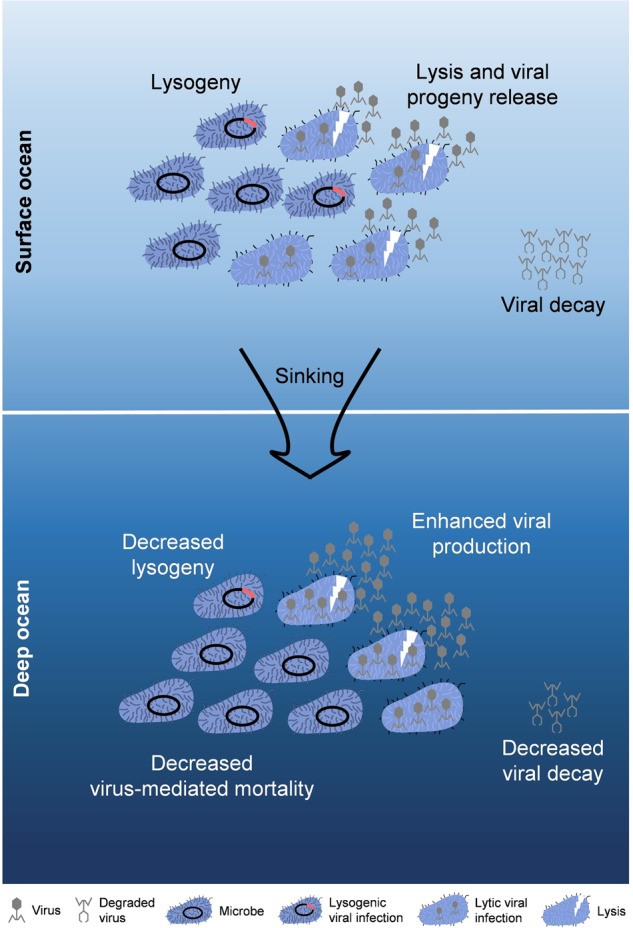
Schematic overview depicting the impact of sinking on viral eco-dynamics and life strategy. When the surface bacterial/viral communities sank into the deep ocean, both lytic and lysogenic VP_R_ significantly increased, which was mainly due to the increased BS, and triggered the switch from the lysogenic to lytic strategy, which was confirmed by the decline in the FLC. However, the VMM and VD_R_ decreased obviously, probably due to cooling inhibition.

Viruses have been found to be beneficial for increasing the abundance, size, and stability of aggregates [83]. Attributed to lysis products and cell residues that acted as biological glue, causing the particles to stick together [65, 84], the release of cell contents by viral lysis could enhance aggregate formation [65, 85]. For example, an investigation suggested that aggregate formation and vertical carbon fluxes were enhanced by the Coccolithovirus infections of coccolithophore, *Emiliania huxleyi*, blooms in the North Atlantic [86]. These indicate that viral infection can “shuttle” surface organic particles into deep waters, and may promote the efficiency of BP in the ocean [87]. In this study, for the first time, we revealed that environmental changes caused by vertical transplantation decreased the VMM, but significantly increased lytic and lysogenic VP by increasing BS and triggering the switch from the lysogenic to lytic strategy to release additional viral particles into the surrounding water (Fig. 4). This could enhance viral productivity, potentially resulting in additional infection and lysis events afterwards. During sinking, such an effect would not only reduce the bacterial consumption of POM by viral lysis but also promote the formation and aggregation of POM by lysis products. This could provide a double-positive feedback for the efficiency of the BP hence increasing the downward flux of organic carbon. However, it is worth noting that the POM-aggregation can also lead to viral decay via irreversible binding, which could be a significant mechanism causing the removal of viruses in the ocean [18, 43, 73]. Hewson and Fuhrman reported that between 20 and 90% of natural marine viruses can be absorbed by POM, dependent on their concentration, size, and source [88]. Therefore, the interactions among particles, bacteria and viruses are complex and further investigations are required to evaluate the net effect of viruses on the efficiency of BP in the ocean.

## Supplementary information

Supplementary information

Figure S1

Figure S2

Figure S3

Table S1

Table S2
