# Supplementary material for: Reduced bacterial mortality and enhanced viral productivity during sinking in the ocean
Source: ISME J. 2022 Apr 1;16(6):1668–75. doi: 10.1038/s41396-022-01224-9 (PMC9123201; doi:10.1038/s41396-022-01224-9)
Supplement: Supplementary file 1 — Supplementary information [file 41396_2022_1224_MOESM1_ESM.docx]

Supplementary Information

Reduced bacterial mortality and enhanced viral productivity during sinking in the ocean

Wei Wei^1,2,3^, Xiaowei Chen^2,4^, Markus G. Weinbauer^5^, Nianzhi Jiao^2,4^*, Rui Zhang^2,4^*

^1^College of the Environment and Ecology, Xiamen University, Xiamen 361102, PR China

^2^State Key Laboratory of Marine Environmental Science, Institute of Marine Microbes and Ecospheres, Xiamen University, Xiamen 361102, PR China

^3^School of Environmental Ecology and Biological Engineering, Wuhan Institute of Technology, Wuhan 430205, PR China

^4^College of Ocean and Earth Sciences, Xiamen University, Xiamen 361102, PR China

^5^Sorbonne Universités, UPMC, Université Paris 06, CNRS, Laboratoire d’Océanographie de Villefranche (LOV), Villefranche-sur-Mer 06230, France.

Corresponding author: Rui Zhang (ruizhang@xmu.edu.cn); Nianzhi Jiao (jiao@xmu.edu.cn)


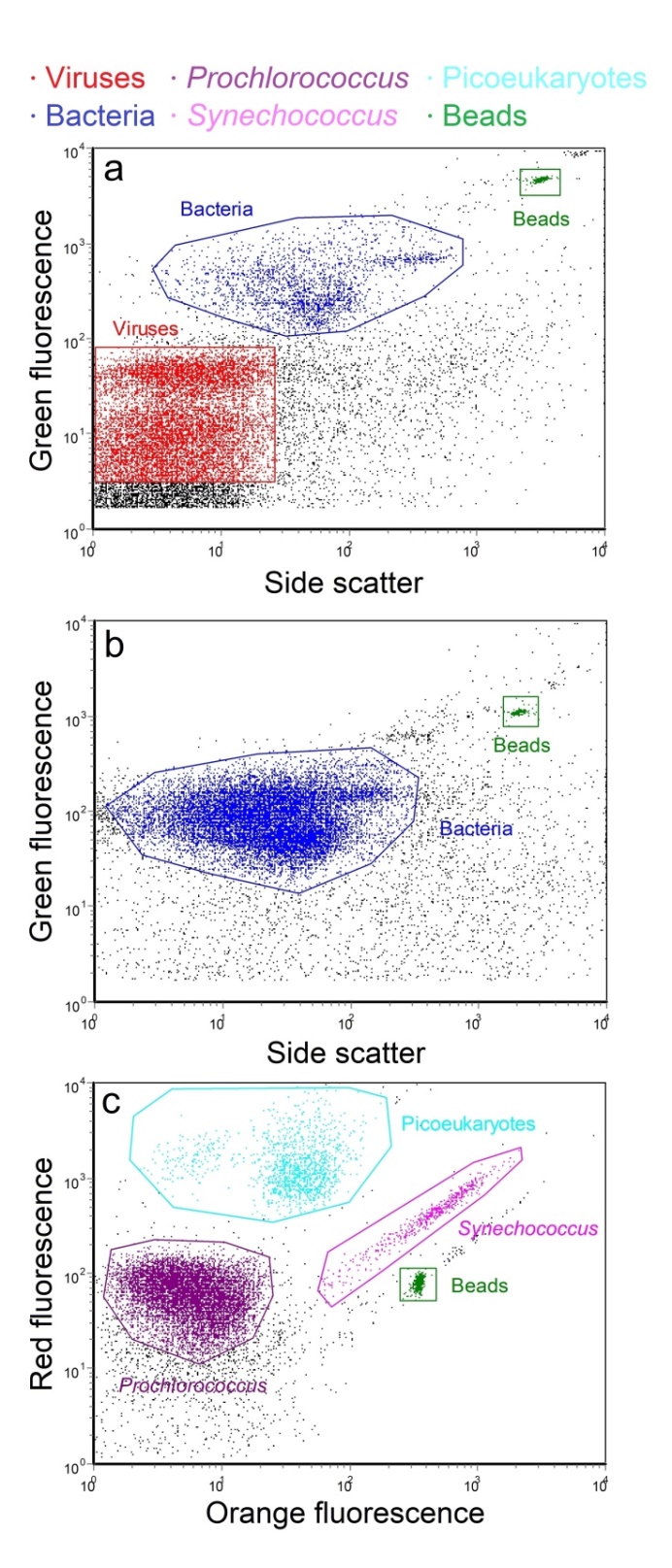


Fig. S1. The scatter plots of flow cytometry analysis for viruses (a), bacteria (a and b) and autotropic microbes (c) (i.e., *Prochlorococcus*, *Synechococcus* and picoeukaryotes) in this study with FCS Express V3 software (De Novo Software, http://www.denovosoftware.com/).


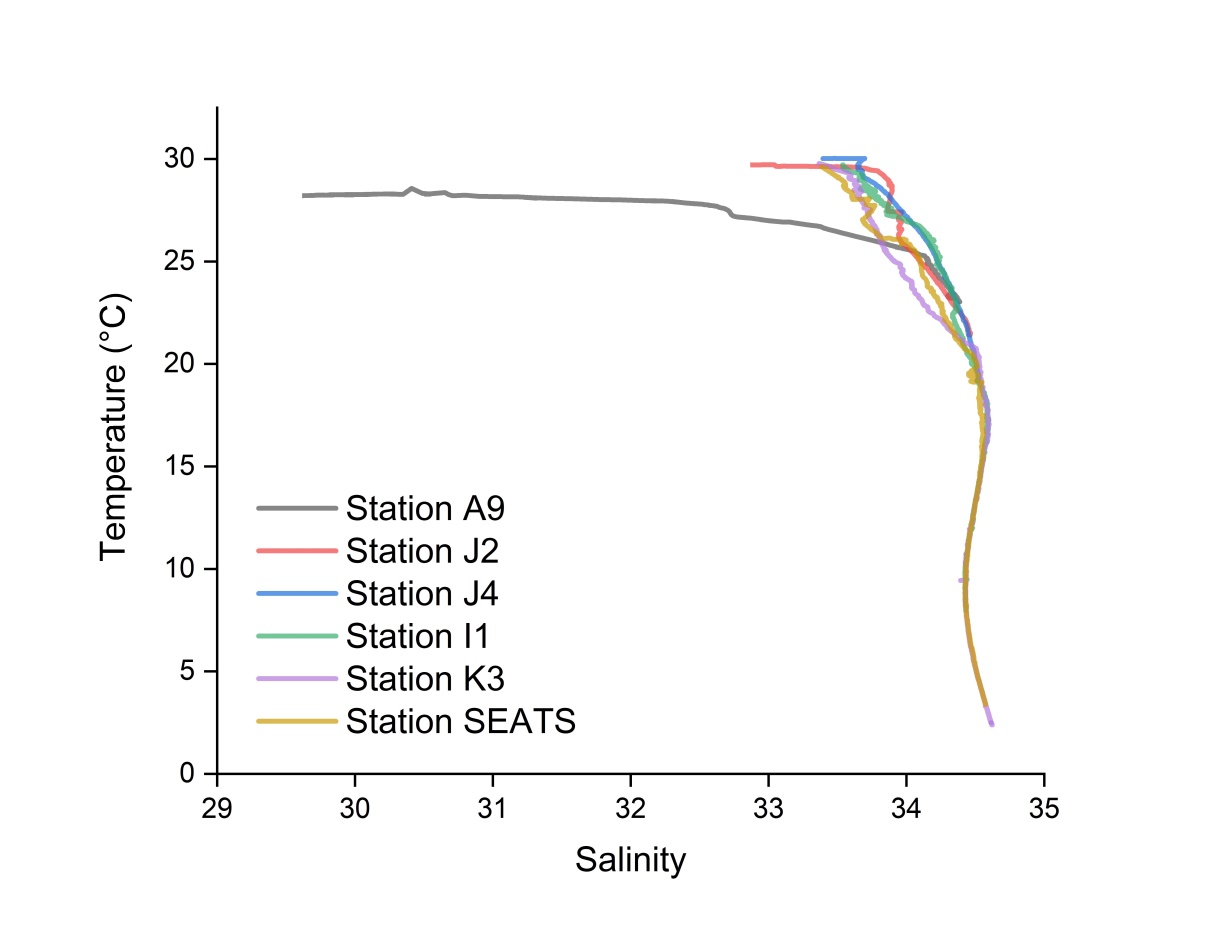


Fig. S2. Temperature versus salinity diagram for the six stations in the SCS.


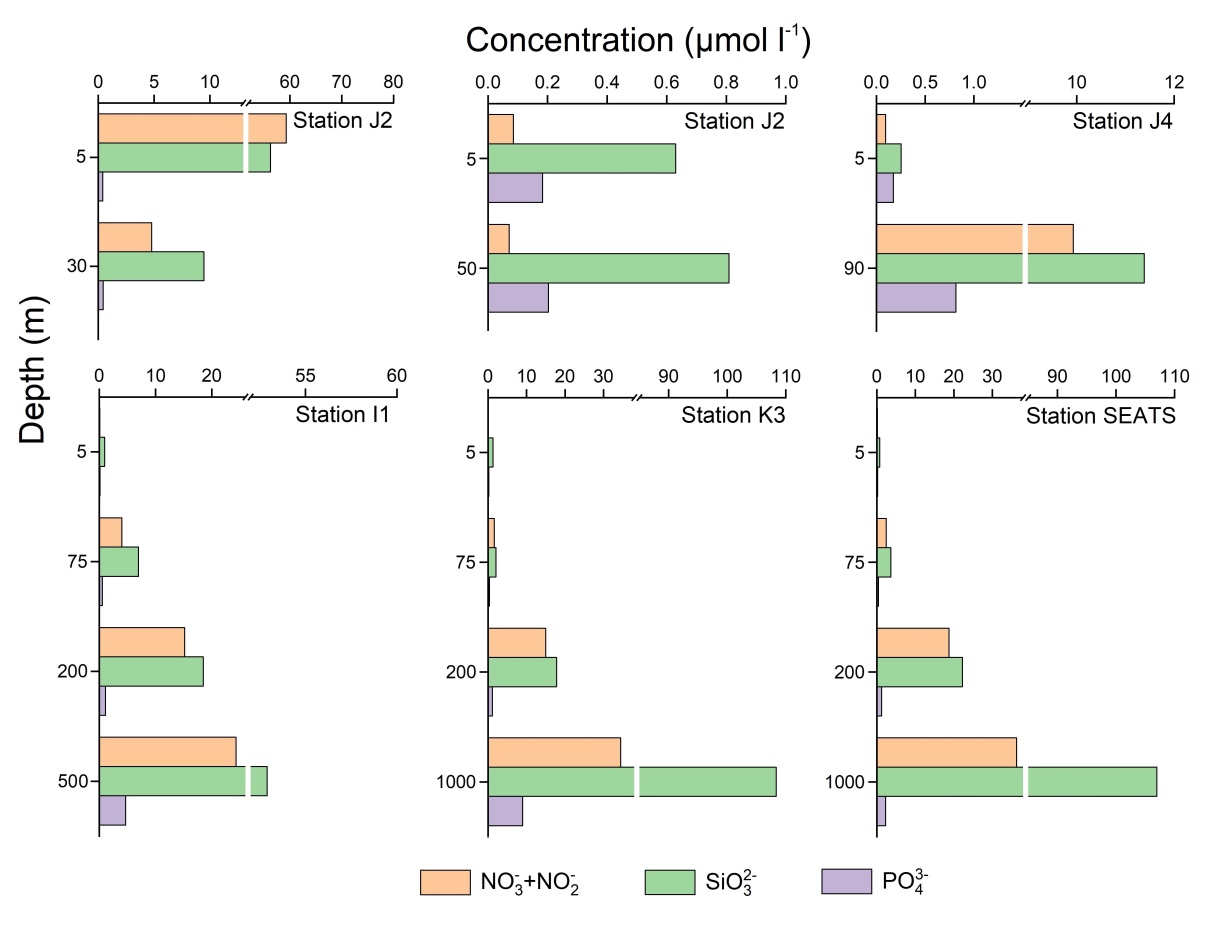


Fig. S3. Vertical distribution of $\text{NO}_{\text{3}}^{\mathbf{-}}$+$\text{NO}_{\text{2}}^{\mathbf{-}}$ (orange), $\text{SiO}_{\text{3}}^{\text{2}\text{–}}$ (green) and $\text{PO}_{\text{4}}^{\text{3}\text{–}}$ (purple) concentrations at the six stations in the SCS.

Table S1. Environmental and biological parameters of the sampling stations with different layers in the SCS. Euk: picoeukaryotic abundance, Syn: *Synechococcus* abundance, Pro: *Prochlorococcus* abundance, Bac: heterotrophic bacterial abundance, VA: viral abundance, VP_R_: viral production rate, VD_R_: viral decay rate, and ND: not detected.

| Stations | Longitude | Latitude | Depth | Temperature | Salinity | $\text{NO}_{\text{3}}^{-}$+$\text{NO}_{\text{2}}^{\text{–}}$ | $\text{SiO}_{\text{3}}^{\text{2–}}$ | $\text{NO}_{\text{2}}^{\text{–}}$ | $\text{PO}_{\text{4}}^{\text{3–}}$ |
| --- | --- | --- | --- | --- | --- | --- | --- | --- | --- |
|  | (°E) | (°N) | (m) | (°C) |  | (μmol l^–1^) | (μmol l^–1^) | (μmol l^–1^) | (μmol l^–1^) |
| A9 | 114 | 22 | 5 | 27.9 | 29.6 | 59.285 | 56.242 | 5.677 | 0.400 |
|  |  |  | 30 | 23.2 | 34.4 | 4.776 | 9.467 | 0.928 | 0.428 |
| J2 | 114.33 | 21.33 | 5 | 29.7 | 33.1 | 0.085 | 0.630 | 0.015 | 0.183 |
|  |  |  | 50 | 24.6 | 34.1 | 0.071 | 0.809 | 0.017 | 0.203 |
| J4 | 114.67 | 20.66 | 5 | 29.7 | 33.5 | 0.093 | 0.254 | 0.013 | 0.173 |
|  |  |  | 50 | 24.7 | 34.3 | 3.371 | 3.962 | 0.159 | 0.415 |
|  |  |  | 90 | 19.1 | 34.6 | 9.932 | 11.388 | 0.043 | 0.815 |
| I1 | 115 | 20 | 5 | 29.7 | 33.5 | 0.087 | 0.956 | 0.013 | 0.160 |
|  |  |  | 75 | 21.9 | 34.4 | 4.014 | 6.983 | 0.038 | 0.544 |
|  |  |  | 200 | 14.2 | 34.5 | 15.184 | 18.495 | 0.034 | 1.116 |
|  |  |  | 500 | 8.5 | 34.4 | 24.303 | 52.901 | ND | 4.691 |
| K3 | 115.5 | 19 | 5 | 29.8 | 33.37 | 0.058 | 1.288 | ND | 0.190 |
|  |  |  | 75 | 22.2 | 34.25 | 1.593 | 2.075 | 0.265 | 0.357 |
|  |  |  | 200 | 15.7 | 34.56 | 14.985 | 17.804 | ND | 1.117 |
|  |  |  | 1000 | 4.4 | 34.53 | 34.465 | 108.358 | ND | 8.949 |
| SEATS | 116 | 18 | 5 | 29.5 | 33.4 | 0.127 | 0.748 | 0.003 | 0.203 |
|  |  |  | 75 | 21.3 | 34.29 | 2.481 | 3.638 | 0.250 | 0.420 |
|  |  |  | 200 | 14.9 | 34.54 | 18.769 | 22.202 | ND | 1.255 |
|  |  |  | 1000 | 4.7 | 34.52 | 36.289 | 106.977 | 0.001 | 2.299 |

| Euk | Syn | Pro | Bac | VA | Lytic VP_R_ | Lysogenic VP_R_ | VD_R_ |
| --- | --- | --- | --- | --- | --- | --- | --- |
| (cells ml^–1^) | (cells ml^–1^) | (cells ml^–1^) | (cells ml^–1^) | (viruses ml^–1^) | (% h^–1^) | (% h^–1^) | (% h^–1^) |
| 6.54 ± 0.69 × 10^3^ | 1.04 ± 0.09 × 10^4^ | 6.30 ± 0.05 × 10^4^ | 1.77 ± 0.07 × 10^6^ | 9.84 ± 0.22 × 10^6^ | 4.30 ± 0.69 | 6.01 ± 2.26 | 1.31 ± 0.11 |
| 1.57 ± 0.34 × 10^3^ | 3.31 ± 0.25 × 10^5^ | 2.48 ± 0.17 × 10^3^ | 1.81 ± 0.04 × 10^6^ | 1.33 ± 0.06 × 10^7^ | 7.51 ± 1.39 | 4.53 ± 0.67 | 0.58 ± 0.10 |
| 3.70 ± 0.32 ×10^3^ | 3.71 ± 0.43 × 10^4^ | 3.23 ± 0.24 × 10^4^ | 1.31 ± 0.15 × 10^6^ | 9.97 ± 0.31 × 10^6^ | 2.94 ± 0.89 | 1.21 ± 0.52 | 1.25 ± 0.06 |
| 3.08 ± 0.47 ×10^3^ | 1.85 ± 0.19 × 10^5^ | 5.22 ± 0.35 × 10^4^ | 1.40 ± 0.09 × 10^6^ | 1.09 ± 0.14 × 10^7^ | 4.01 ± 1.72 | 4.02 ± 0.88 | 1.16 ± 0.51 |
| 8.63 ± 1.93 × 10^2^ | 6.39 ± 0.62 × 10^4^ | 1.57 ± 0.08 × 10^3^ | 9.64 ± 0.10 × 10^5^ | 1.01 ± 0.01 × 10^7^ | 4.19 ± 2.11 | 12.28 ± 1.91 | 2.68 ± 0.06 |
| 4.60 ± 0.65 × 10^3^ | 3.86 ± 0.19 × 10^4^ | 1.02 ± 0.03 × 10^5^ | 1.04 ± 0.03 × 10^6^ | 8.45 ± 0.69 × 10^6^ | 7.56 ± 3.43 | 10.16 ± 4.56 | 1.89 ± 0.28 |
| 9.37 ± 4.05 × 10^2^ | 2.18 ± 0.14 × 10^4^ | 1.33 ± 0.07 × 10^4^ | 5.45 ± 0.16 × 10^5^ | 3.22 ± 0.12 × 10^6^ | 6.21 ± 3.69 | 5.09 ± 1.67 | 2.05 ± 0.17 |
| 1.51 ± 0.43 × 10^2^ | 9.23 ± 0.70 × 10^4^ | 3.94 ± 0.43 × 10^2^ | 7.21 ± 0.02 × 10^5^ | 9.70 ± 0.19 × 10^6^ | 2.47 ± 1.43 | 7.03 ± 0.47 | 4.14 ± 0.50 |
| 3.12 ± 0.30 × 10^3^ | 3.41 ± 0.21 × 10^4^ | 3.93 ± 0.25 × 10^4^ | 4.44 ± 0.18 × 10^5^ | 4.42 ± 0.12 × 10^6^ | 31.12 ± 6.34 | 3.83 ± 0.69 | 3.83 ± 1.24 |
| 0.91 ± 1.28 × 10^2^ | 2.31 ± 0.01 × 10^4^ | 4.24 ± 2.57 × 10^2^ | 2.51 ± 0.04 × 10^5^ | 1.67 ± 0.01 × 10^6^ | 9.75 ± 0.74 | 2.77 ± 1.09 | 2.27 ± 0.68 |
| ND | 4.56 ± 0.34 × 10^4^ | 9.10 ± 4.30 × 10^1^ | 1.47 ± 0.19 × 10^5^ | 1.00 ± 0.01 × 10^6^ | 10.19 ± 2.80 | 5.01 ± 1.22 | 2.86 ± 0.39 |
| 9.51 ± 2.34 × 10^2^ | 4.88 ± 0.13 × 10^4^ | 1.48 ± 0.13 × 10^3^ | 8.03 ± 0.28 × 10^5^ | 7.55 ± 0.14 × 10^6^ | 7.39 ± 1.28 | 3.51 ± 1.55 | 1.18 ± 0.21 |
| 3.80 ± 0.27 × 10^3^ | 3.04 ± 0.03 × 10^4^ | 6.19 ± 0.19 × 10^4^ | 5.54 ± 0.22 × 10^5^ | 7.50 ± 0.27 × 10^6^ | 2.28 ± 0.12 | 4.31 ± 0.64 | 0.77 ± 0.04 |
| 4.50 ± 2.50 × 10^1^ | 2.82 ± 0.04 × 10^4^ | 4.99 ± 1.51 × 10^2^ | 1.87 ± 0.03 × 10^5^ | 2.01 ± 0.12 × 10^6^ | 1.92 ± 0.34 | 3.98 ± 0.72 | 1.05 ± 0.01 |
| 1.50 ± 2.10 × 10^1^ | 2.41 ± 0.01 × 10^4^ | 2.72 ± 0.80 × 10^2^ | 1.65 ± 0.01 × 10^5^ | 7.88 ± 0.09 × 10^5^ | 6.16 ± 0.62 | 4.51 ± 0.55 | 0.9 ± 0.35 |
| 8.18 ± 0.43 × 10^2^ | 3.41 ± 0.06 × 10^4^ | 1.97 ± 0.64 × 10^2^ | 8.82 ± 0.39 × 10^5^ | 7.41 ± 0.15 × 10^6^ | 7.54 ± 0.91 | 5.79 ± 2.22 | 2.01 ± 0.10 |
| 4.03 ± 0.49 ×10^3^ | 1.15 ± 0.12 × 10^4^ | 1.08 ± 0.03 × 10^5^ | 4.84 ± 0.03 × 10^5^ | 1.16 ± 0.02 × 10^7^ | 3.73 ± 1.97 | 8.89 ± 1.35 | 1.17 ± 0.06 |
| 1.50 ± 3.60 × 10^1^ | 1.30 ± 0.05 × 10^4^ | 3.63 ± 1.28 × 10^2^ | 1.82 ± 0.17 × 10^5^ | 1.75 ± 0.15 × 10^6^ | 4.59 ± 1.53 | 9.52 ± 1.78 | 1.35 ± 0.05 |
| ND | 2.25 ± 0.06 × 10^4^ | 1.81 ± 0.43 × 10^2^ | 7.68 ± 2.70 × 10^4^ | 5.91 ± 0.37 × 10^5^ | 22.73 ± 5.98 | 14.81 ± 3.43 | 0.95 ± 0.39 |

Table S2. Results of the multivariate regression analysis with forward selection (DISTLM-*forward*) to explain the variability in the VBR in the SCS.

| Variable | Pseudo-F | *p* level | Explained variance | Cumulative variance |
| --- | --- | --- | --- | --- |
| Viral abundance | 85.99 | 0.00 | 0.52 | 0.52 |
| $\mathrm{PO}_{4}^{3-}$ concentration | 5.82 | 0.02 | 0.03 | 0.55 |
| *Synechococcus* abundance | 5.14 | 0.03 | 0.02 | 0.57 |
