# Supplementary figures and images for: Reduced bacterial mortality and enhanced viral productivity during sinking in the ocean
Source: ISME J. 2022 Apr 1;16(6):1668–75. doi: 10.1038/s41396-022-01224-9 (PMC9123201; doi:10.1038/s41396-022-01224-9)

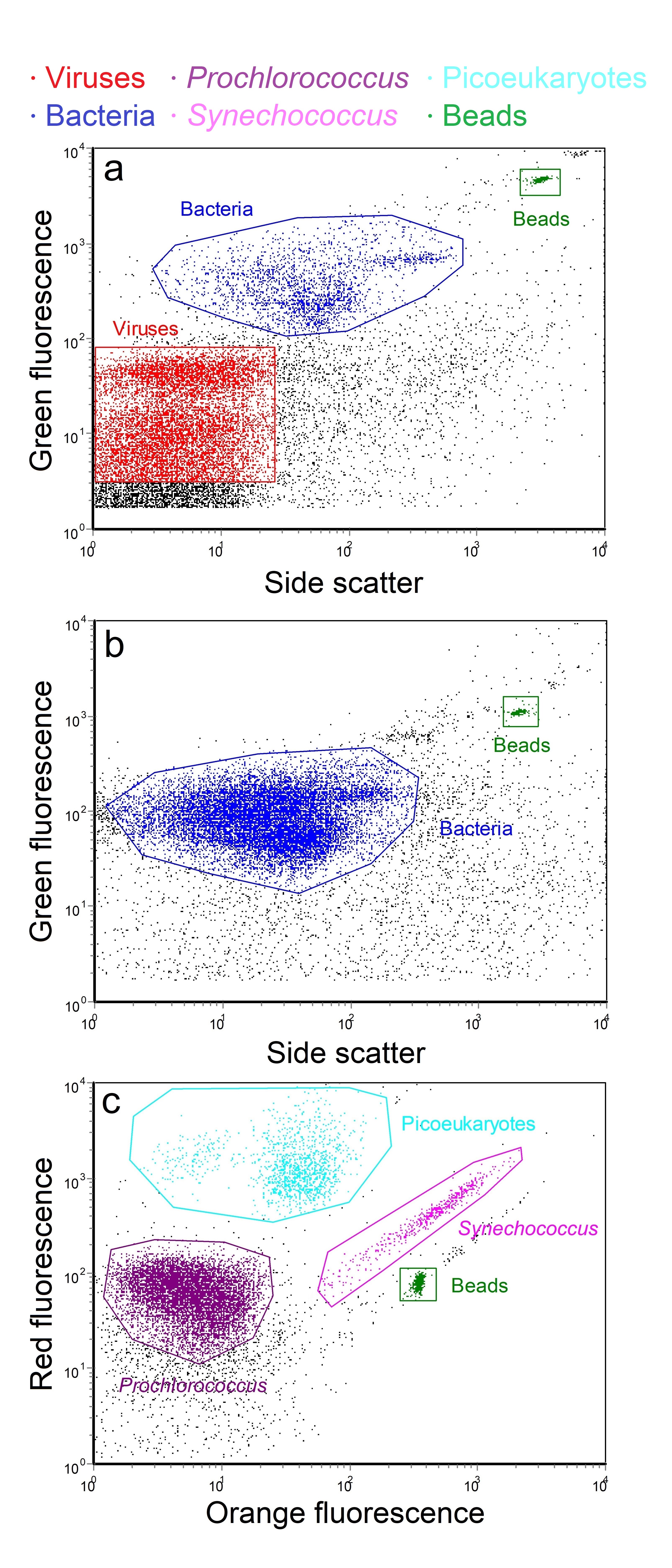

Supplement: Supplementary file 2 — Figure S1 [file 41396_2022_1224_MOESM2_ESM.jpg]

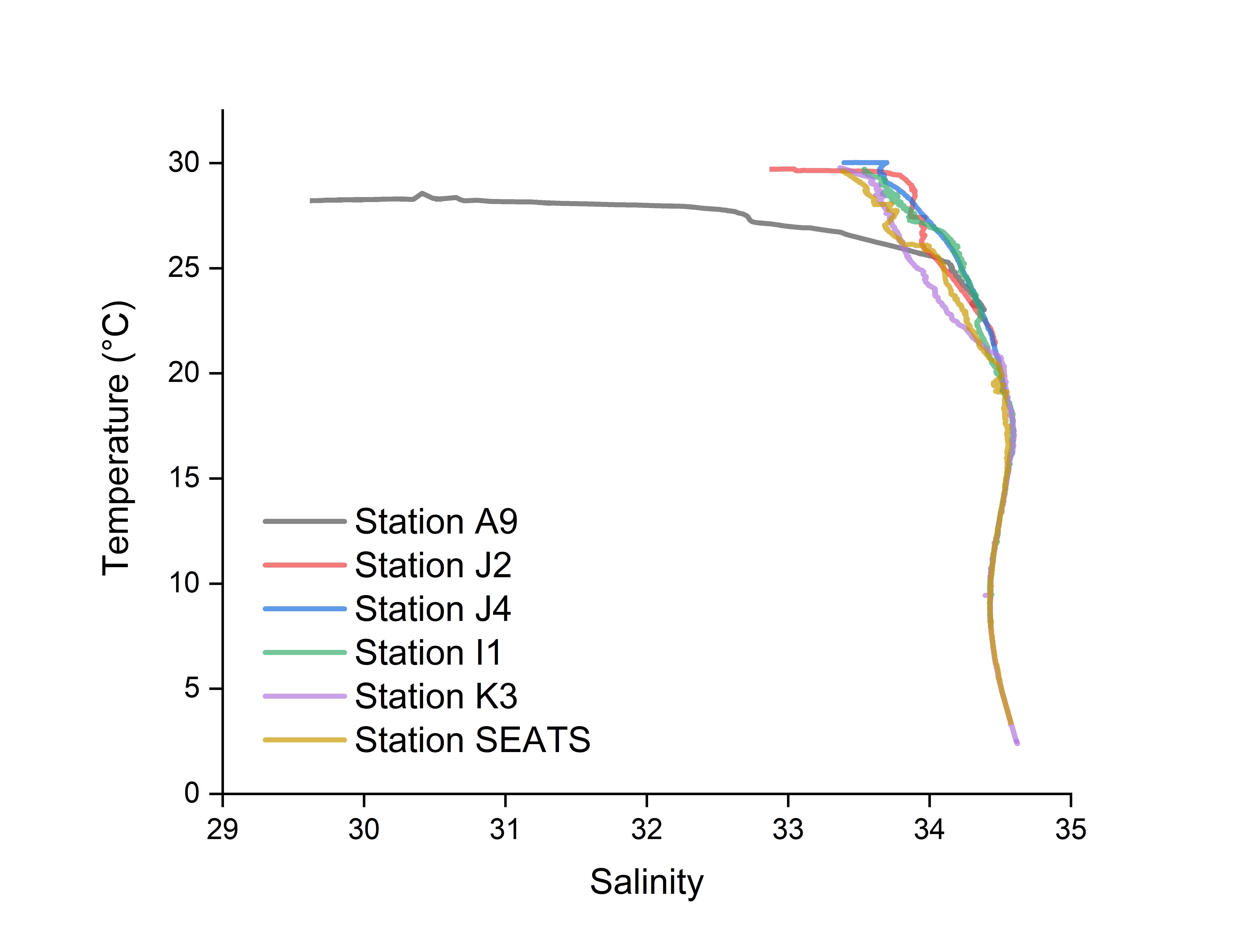

Supplement: Supplementary file 3 — Figure S2 [file 41396_2022_1224_MOESM3_ESM.jpg]

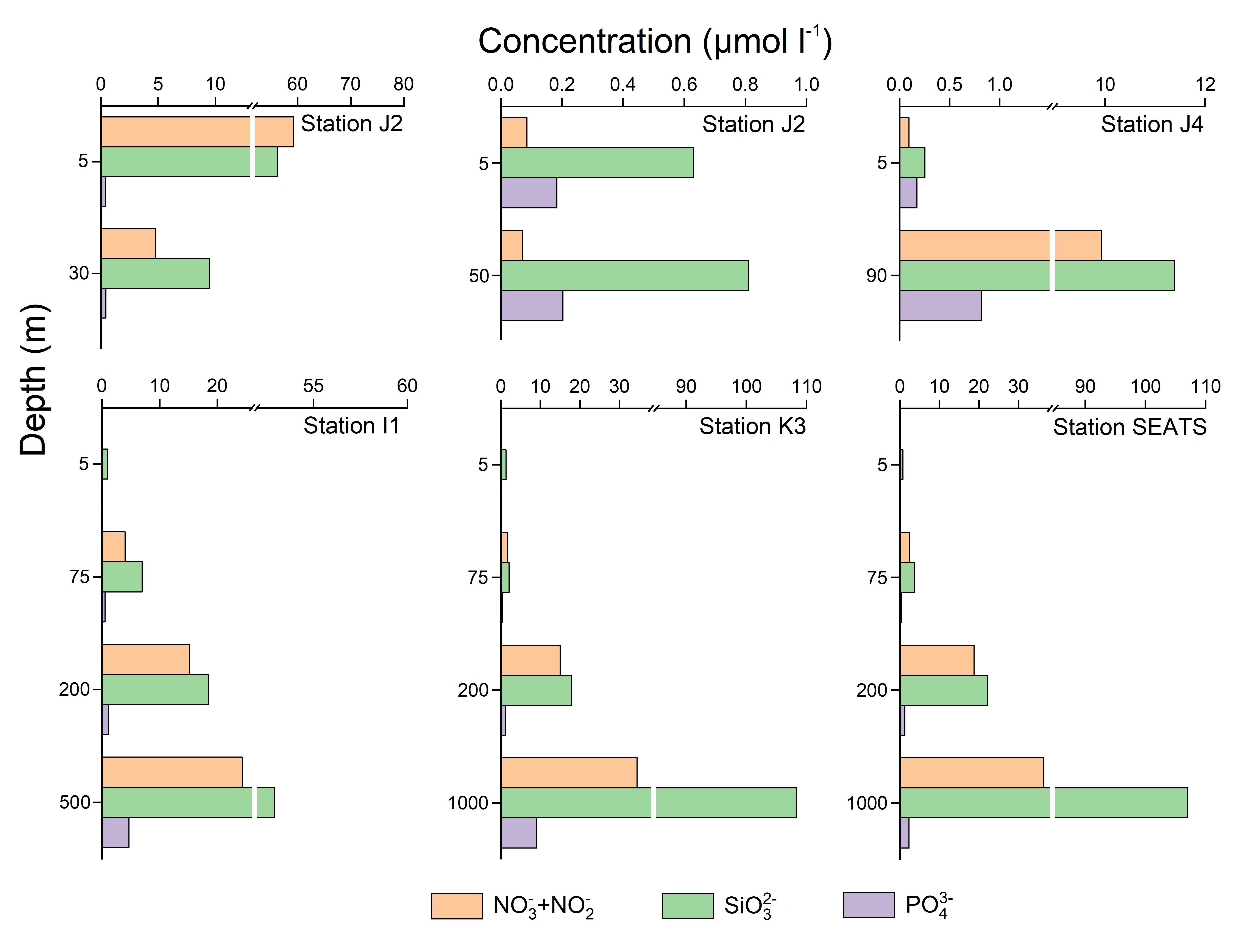

Supplement: Supplementary file 4 — Figure S3 [file 41396_2022_1224_MOESM4_ESM.jpg]
